# Men’s Knowledge of and Behavior toward Mammography Screening: A Cross-Sectional Study

**DOI:** 10.31557/APJCP.2021.22.7.2251

**Published:** 2021-07

**Authors:** Rawan U. Gadi, Leena A. Merdad, Nada J. Farsi, Rolina K. Al-Wassia

**Affiliations:** 1 *Department of Dental Public Health, Faculty of Dentistry, King Abdulaziz University, Jeddah, Saudi Arabia. *; 2 *Department of Radiology, Faculty of Medicine, King Abdulaziz University, Jeddah, Saudi Arabia. *

**Keywords:** Male, humans-female, cross-sectional studies, mammography, early detection of cancer

## Abstract

**Background:**

Breast cancer (BC) is a public health problem that affects many populations worldwide. Women’s health care behavior, including seeking mammography screening, might be affected by men, especially in conservative Arab societies. Few studies have investigated men’s behavior toward mammography for female relatives. The main aims of this study were (i) to evaluate men’s knowledge about mammography screening and (ii) to assess men’s behavior toward women regarding mammogram screening and the factors influencing their behavior.

**Methods:**

A cross-sectional study was conducted among male residents of the five main geographic areas of Saudi Arabia. Data were obtained with a self-administered questionnaire. In addition to sociodemographic data, the questionnaire assessed respondents’ general knowledge about mammograms, their behavior toward female family members who use mammography, and their perceptions about awareness campaigns.

**Results:**

A total of 9691 male respondents were included in the study. The majority (79%) recommended mammography to their female family members. Multiple factors were significantly associated with men recommending mammograms, including age (P<0.01), education (P<0.01), employment status (P<0.01), and region (P<0.01). Only 33.8% of the participants had a high knowledge score about mammography. Approximately 45% of respondents reported that BC awareness campaigns were weak, while 48% were not aware of BC screening programs.

**Conclusions:**

Despite their positive behavior in recommending mammograms to female relatives, men exhibited a notable lack of knowledge about mammography. Establishing national programs and educational campaigns for men to explain the benefits of screening and access to free mammography are essential.

## Introduction

Breast cancer (BC) is a significant public health problem that affects many populations worldwide. It is the most frequently diagnosed cancer among women, with an estimated 1.67 million new cases reported in 2012 (Ferlay et al., 2015). It is the fifth cause of death among all cancers and the primary cause of mortality among women in developing countries (Ferlay et al., 2015). Late-stage discovery of BC has been considered the main reason for high mortality rates among women in many developing countries, and it is most often associated with a lack of BC screening (Bhikoo et al., 2011). Screening mammography has been proven to allow early detection of BC, thus reducing mortality (Gøtzsche and Jørgensen, 2013). The National Comprehensive Cancer Network recommends annual mammogram screening for women aged 40 years and older (Bonaccio et al., 2019). In Saudi Arabia, the Ministry of Health (MOH) recommends regular mammograms every 2 years for all women aged 40-50 years and regular mammograms once every 1-2 years for all women aged 50-69 years. In addition, MOH recommends that all women with a family history of BC have mammogram screening 10 years before the age of the patient with BC in the family (Saudi Ministry of Health).

Social support provided by families plays a key role in guiding and encouraging women to follow BC early detection recommendation (Petro-Nustus and Mikhail, 2002; Soskolne et al., 2006; Lamyian et al., 2007; Donnelly et al., 2013; Taha et al., 2013). Men play a vital role in conservative societies, such as those in Arab countries; their advice and support in encouraging their female relatives to seek breast health care and screening services has been linked to enhancement of BC screening practices (Donnelly et al., 2013). In addition, receiving social support and encouragement, especially from one’s spouse, could positively influence a woman’s decision to have a mammogram (Donnelly et al., 2013). However, the conservative nature of some societies still prevents men from directly discussing sensitive issues with their female family members (Al Dasoqi et al., 2017). American Indian men, for instance, believe that BC is a woman’s issue and they do not talk about women’s body parts or mammography (Filippi et al., 2014). Thus, it is important to assess the knowledge and behavior of men regarding mammography screening of female family members to encourage women to take better care of their health and participate in screening.

The vast majority of studies conducted on BC screening among men have explored their level of knowledge and their attitudes toward mammography; however, few studies have assessed their behavior toward women regarding mammogram screening. A study conducted in Switzerland demonstrated that men were almost as knowledgeable as women about mammography screening and had more positive attitudes toward mammography than women did (Chamot and Perneger, 2002). Another study conducted in Poland found that 72% of male participants recognized that a mammogram was the best method for detecting BC, and 66% thought that fear and fear of disease were the factors that prevented patients from seeking mammograms (Gøtzsche and Jørgensen, 2013).

In the Arab world, a qualitative study conducted in Qatar among men concluded that most of the participants had a basic knowledge of BC screening activities and felt they had a vital role in encouraging women to participate in these activities (Donnelly et al., 2017). A study that explored Jordanian men’s perspectives on BC and breast health showed that they felt they had an instrumental role in supporting and encouraging their wives to follow BC early detection recommendations (Taha et al., 2013). On the other hand, another study conducted in Jordan found that male college students showed poor knowledge about BC screening and uncertainty about the role of men in screening (Al Dasoqi et al., 2017). In Saudi Arabia, a number of studies have shown a low level of knowledge regarding BC and the importance of mammogram screening (Ravichandran et al., 2011; Al-Amoudi et al., 2012; Al-Amoudi and Abduljabbar, 2012; Al-Amoudi et al., 2016). However, in a study conducted among male high school students in Saudi Arabia, approximately 79% of the participants identified “persuasion of female relatives to have mammograms” and 71% identified “psychological support” as the main roles of men regarding BC. Only 46.8% stated that they would accompany their female relatives when having mammograms, however (Al-Amoudi et al., 2016). Only one study explored the behavior of men toward women regarding mammogram screening and found that males would recommend screening to their female relatives (Al-Amoudi et al., 2016).

Ensuring the continuous development of effective breast health promotion strategies to increase mammography use requires increased knowledge, support, and involvement of men to capitalize on family support. Therefore, a better understanding of men’s level of knowledge about mammography, as well as their behavior about recommending it to relatives, is essential. Few studies have focused on the behavior of men toward the mammography practice of their female relatives in general, and fewer have done so in Saudi Arabia in particular. The main objectives of the current study were (i) to evaluate men’s knowledge about mammography screening and (ii) to assess men’s behavior toward female relatives regarding mammogram screening and the factors influencing their behavior.

## Materials and Methods

*Study design and subjects*

This study was part of a larger cross-sectional study designed to assess knowledge of and behavior toward BC and mammography practice among 40895 male and female residents of the following geographic areas of Saudi Arabia: (i) Central (Riyadh, Al-Qassim, Ha’il), (ii) Western (Mecca, Madinah), (iii) Northern (Tabouk, Al-Jawf, Northern Border), (iv) Southern (Al-Bahah, Aseer, Jazan, Najran), and (v) Eastern regions (Al-Wassia et al., 2017). Only male participants were included in the current study (n=9,691).

*Questionnaire and data collection*

The questionnaire was compiled from a validated questionnaire used in a similar previous study (Hagi et al., 2013), and additional relevant questions were added. Experts from various medical fields pertinent to BC, radiation oncology, medical oncology, radiology, and surgery provided feedback about the questionnaire. The questionnaire was composed of four sections: (i) sociodemographic characteristics; (ii) knowledge about BC, its symptoms, and risk factors; (iii) mammography knowledge and recommendation to female family members (mother, wife, or daughter); and (iv) perceptions about BC awareness and campaigns in Saudi Arabia. 

Knowledge about mammography was measured by asking men three questions about mammography practice: (i) Do you think that the mammogram is the best method for breast cancer diagnosis?; (ii) At what age should a woman get a mammogram?; (iii) and How often should a woman get a mammogram in the suitable age range?. Responses to knowledge questions were categorized as correct or incorrect and assigned a score of 1 for correct answers and 0 for “don’t know” or incorrect answers. A total knowledge score was calculated by summing the scores of the questions of each participant, with the final scores ranging from 0 to 3. This total knowledge score was then categorized as either low knowledge (scores 0 and 1) or high knowledge (scores 2 and 3). Participants’ recommendations to immediate family relatives regarding a mammogram were assessed with the following question: “Have you ever recommended a mammogram to a female family member (mother, wife, or daughter)?”

Perceptions about BC awareness and campaigns were assessed in the following multiple choice questions: “Do you think that there are enough awareness campaigns in Saudi Arabia?”; “How would you rate the awareness of our society in general about breast cancer?”; and “Are you aware of any breast cancer screening programs in Saudi Arabia?”

The questionnaire was initially written in English. It was then translated to Arabic by using the forward-backward-forward technique (Al-Wassia et al., 2017). The questionnaire was provided to participants in both Arabic and English, and they chose the language in which they preferred to complete the survey. 

A convenience sample of men residing in the five geographic regions was selected. In each of these regions, electronic questionnaires were distributed and promoted through several social media sites and applications. To ensure broad distribution of the questionnaires, especially to individuals with limited access to social media, we distributed paper-based questionnaires in public venues such as local shopping malls in each geographic region. Data were collected from February 2015 to May 2015.

*Statistical analysis*

Frequencies and percentages were used for the sociodemographic characteristics of the participants and for responses to general knowledge questions about mammography. The two main outcomes in the study were (i) whether participants recommended mammography to immediate female family members (mother, wife, or daughter) (yes/no) and (ii) mammography knowledge scores (low/high). The associations between sociodemographic variables and the two outcomes were assessed by using the chi-square test. Responses to questions about BC awareness and campaigns were reported as frequencies and percentages. Statistical analyses were conducted with Stata 12.1 (StataCorp LP, College Station, Texas, USA).

## Results

The characteristics of the 9691 male respondents included in this study are displayed in Table 1. Fifty-seven percent were employed, and 38% had an income of 9,000-15,000 SAR. Forty-one percent had a university degree, and an equivalent proportion completed only high school or attained a diploma. Twenty-eight percent of participants were from the Western province, 20% from each of the Central and Southern provinces, 17% from the Northern province, and 15% from the Eastern province. The majority of the respondents had no previous family history of BC (71%).

One-third of the respondents reported that at least one of their immediate female family members had received a mammogram (n=2844), 26% reported that their female family members had not received a mammogram, and 44% did now know whether their female family members had received one or not. Regarding mammography knowledge, approximately 44% of the subjects knew that mammograms should be performed every 1-2 years for the suitable age range, 38% recognized that the mammogram is the best method for cancer diagnosis, and only 25% recognized that the age at which women should start getting a mammogram is 40 years. When participants were asked to rate their perceived knowledge about mammography, the majority reported it to be poor (58%) and 19% reported it to be fair. A low proportion of the participants reported it to be excellent or very good (12% each) (data not shown). 

The associations between sociodemographic variables and whether participants recommended mammography to their female family members are presented in Table 2. Most of the participants recommended mammography (79%). Participants over 30 years old were more likely to recommend mammography than were younger participants. Participants with a university degree or higher and those employed were also more likely to recommend mammography to family members than were participants with lower educational levels and those who were unemployed or retired. Among residents from the Eastern, Western, and Southern regions, 83%, 82%, and 80% recommended mammography, respectively; of the respondents in the Central and Northern regions, 79% and 71% recommended mammography, respectively. Of the participants with a high mammography knowledge score, 88% recommended mammography, whereas 77% of those with a low knowledge score did so.

Table 3 demonstrates the associations between sociodemographic variables and knowledge scores. The percentage of participants with high mammography knowledge scores significantly increased with increasing age, educational level, and income; high mammography knowledge was also significantly higher among retired subjects in comparison to those with other employment conditions. Regarding geographic areas, 41% of the Eastern region respondents and 37% of respondents from the Western and Central regions had high mammography knowledge scores, whereas only 27% of Southern and 26% of Northern area respondents had high knowledge scores. Respondents with a famihistory of BC had a higher percentage of high knowledge scores (44%) than did respondents without a family history (33%).

Regarding participants’ perceptions about BC awareness campaigns, only 37% of respondents thought that there are enough cancer awareness campaigns in Saudi Arabia, and 45% reported that these campaigns are weak. Only 12% thought that these awareness campaigns are excellent, and 46% thought that they are of average quality. In addition, 81% were not aware of BC screening programs in the country, compared with 19% who were aware (data not shown).

**Table 1:** Sociodemographic Characteristics of Participants

Sociodemographic variable	N (%)
Total	9,691
Age (y)	
<20	1,929 (20)
21-30	3,849 (40)
31-40	2,359 (24)
>40	1,536 (16)
Occupation	
Unemployed	3,729 (39)
Employed	5,505 (57)
Retired	348 (4)
Education	
Less than high school	795 (8)
High school/diploma	3,940 (41)
University	3,982 (41)
Postgraduate	920 (10)
Family income (SAR/month)	
<8,000	2,730 (29)
9,000-15,000	3,618 (38)
16,000-25,000	2,050 (22)
>26000	1,136 (12)
Region	
Western	2,705 (28)
Eastern	1,401 (15)
Central	1,944 (20)
Northern	1,622 (17)
Southern	1,970 (20)
Family history of breast cancer	
Yes	1,532 (16)
No	6,712 (71)
I do not know	1,271 (13)

SAR, Saudi Arabian Riyal

**Table 2:** Associations between Participants’ Sociodemographic Characteristics and Recommending Mammography to Female Family Members

Sociodemographic Variable	Total N	Ever recommended a mammogram N (%)	Never recommended mammogram N (%)	P-value^a^
Total	9,318	7,392 (79)	1,926 (21)	---
Age (y)				
<21	1,808	1,326 (73)	482 (27)	<0.001
21-30	3,724	2,935 (79)	789 (21)	
31-40	2,287	1,915 (84)	372 (16)	
>40	1,482	1,204 (81)	278 (19)	
Education				
Less than high school	728	494 (68)	234 (32)	<0.001
High school/diploma	3,753	2,865 (76)	888 (24)	
University	3,896	3,228 (83)	668 (17)	
Postgraduate	897	772 (86)	125 (14)	
Occupation				
Not employed	3,569	2,732 (77)	837 (23)	<0.001
Employed	5,320	4,326 (81)	994 (19)	
Retired	335	264 (79)	71 (21)	
Family income (SAR/month)				
<8,000	2,637	2,056 (78)	581 (22)	0.07
9,000-15,000	3,487	2,785 (80)	702 (20)	
16,000-25,000	1,958	1,551 (79)	407 (21)	
>26,000	1,098	896 (82)	202 (18)	
Region				
Western	2,618	2,146 (82)	472 (18)	<0.001
Eastern	1,370	1,136 (83)	234 (17)	
Central	1,925	1,528 (79)	397 (21)	
Northern	1,471	1,038 (71)	433 (29)	
Southern	1,888	1,510 (80)	378 (20)	
Family history of breast cancer				0.666
Yes	1,471	1,194 (81)	277 (19)	
No	6,524	5,327 (82)	1,197 (18)	
Knowledge score				<0.001
Low	5,384	4,151 (77.1)	1,233 (22.9)	
High	2,813	2,468 (87.7)	345 (12.3)	

SAR, Saudi Arabian Riyal; ^a^Chi-square test used

**Table 3:** Association between Participants’ Sociodemographic Characteristics and Their Mammography Knowledge Scores

Sociodemographic variable	Total N	Low score N (%)	High score N (%)	P-value
Total	8,406	5,566 (66.2)	2,840 (33.8)	---
Age (y)				
<21	1,453	1,081 (74)	372 (26)	<0.001
21-30	3,425	2,298 (67)	1,127 (33)	
31-40	2,133	1,409 (66)	724 (34)	
>40	1,383	769 (56)	614 (44)	
Education				
Less than high school	616	468 (76)	148 (24)	<0.001
High school/diploma	3,252	2,304 (71)	948 (29)	
University	3,627	2,313 (64)	1,314 (36)	
Postgraduate	870	451 (52)	419 (48)	
Occupation				
Not employed	3,075	2,080 (68)	995 (32)	<0.001
Employed	4,945	3,251 (66)	1,694 (34)	
Retired	310	177 (57)	133 (43)	
Family income (SAR/month)				
<8,000	2,302	1,655 (72)	647 (28)	<0.001
9,000-15,000	3,187	2,196 (69)	991 (31)	
16,000-25,000	1,777	1,073 (60)	704 (40)	
>26,000	1,028	565 (55)	463 (45)	
Region				
Western	2,423	1,537 (63)	886 (37)	<0.001
Eastern	1,220	724 (59)	496 (41)	
Central	1,791	1,135 (63)	656 (37)	
Northern	1,190	878 (74)	312 (26)	
Southern	1,742	1,263 (73)	479 (27)	
Family history of breast cancer				
Yes	1,352	757 (56)	595 (44)	<0.001
No	6,019	4,008 (67)	2,011 (33)	

SAR, Saudi Arabian Riyal

## Discussion

BC is considered the most common malignancy among women worldwide and has tremendous implications for general health, emotional status, and socioeconomic status (Hortobagyi et al., 2005; Azubuike et al., 2018). In the current study, we aimed to investigate men’s knowledge of and behavior toward mammography screening for their female relatives. Most subjects showed positive behaviors toward mammography screening, as 79% of them recommended it for their female relatives; they demonstrated, however, poor knowledge about mammography. Older age, higher education levels, and being employed were significantly associated with men recommending mammography. The percentage of participants with high mammography knowledge scores significantly increased with a family history of BC and increasing age, educational level, and income. These findings are of paramount importance and call for further attention to raise BC awareness among men, as they play a vital role in Arab women’s lives (Remennick, 2006; Taha et al., 2013). It is crucial to explore men’s knowledge of and behavior toward mammography to help increase BC screening rates among women in order to improve health outcomes.

Poor knowledge of breast screening practices plays a major role in the delay of BC detection (Mamdouh et al., 2014). The findings of the current study revealed that participants lacked sufficient knowledge about mammography screening, as only a quarter of the participants in our study were able to report the correct age for women to start mammogram examination, and only 38% identified mammography as the best screening method for BC diagnosis. Similarly, other investigators have reported a low level of knowledge about mammograms among men. In a previous study targeting Saudi males to assess their knowledge about BC, approximately 26% of respondents recognized mammography as the best tool for early BC detection (Al-Amoudi and Abduljabbar, 2012). Another study was conducted among Saudi male medical students, who were expected to be more aware of BC screening. However, only 17.2% knew what a mammogram was (Al-Amoudi et al., 2012). This low level of knowledge among men could be attributed to sociocultural factors: Jordanian males, for instance, described BC as a women’s issue in a qualitative study among Jordanian males (Al Dasoqi et al., 2017).

Interestingly, and despite the noticeable lack of knowledge about mammography, our findings demonstrated that 79% of participants recommended mammography for their female family members. This positive behavior of men toward mammography is in agreement with previous findings from a local study conducted among male high school students, as 79.4% identified the role of men in persuading relatives and others to have mammograms (Al-Amoudi et al., 2016). This conflicting finding in our study suggests that men’s education about mammogram screening is important. Moreover, this behavior is promising since, in conservative societies such as those of Arab countries, major sociocultural barriers impede screening and early detection of BC (Donnelly et al., 2013). In these societies, men have a major impact on women’s decision making regarding health care services and self-care perceptions (Taha et al., 2013). In addition, men contribute to shaping the social norms regarding BC screenings (Chamot and Perneger, 2002). Therefore, the assessment of men’s knowledge and attitudes about BC and mammography in targeted geographic areas is a prerequisite for designing suitable awareness programs and activities to increase the involvement of men in early detection of female BC.

Our findings also indicate that several sociodemographic characteristics were significantly associated with better knowledge scores: age, education, occupation, income, region, and family history of BC. In fact, we found that older age and higher levels of education are significantly associated with higher knowledge scores, and this finding is consistent with similar results reported among Saudi women, where a higher education level was a significant positive predictor of better knowledge about mammography screening (Al-Wassia et al., 2017). This could be attributed to the wider experience and high level of awareness about the importance of mammography among older and highly educated individuals. A family history of BC was also found to be significantly associated with better knowledge. Similar findings were reported in a study conducted among Saudi women that demonstrated that having a positive history of BC among relatives was a significant positive predictor of knowledge (Al-Wassia et al., 2017). These results suggested that having a positive family history of BC could increase one’s awareness of the disease and lead women to seeking preventive practices.

In the present study, several factors such as age, education, and occupation were significantly associated with men recommending mammography for female relatives. Older and more educated participants were more inclined to recommend screening to female relatives. The relationship between education and positive behavior toward mammography shows that enhanced knowledge among men may lead to more support for women to be screened, as health knowledge is gained through education and reflected in attitudes and behavior. Furthermore, employed participants appeared to be more likely to encourage health-related behaviors, such as recommending mammography screening. This might be explained by the link between employment status and health (Hobson, 2007).

Regarding the perception of the participants about BC awareness and campaigns in Saudi Arabia, our study demonstrated that only 37% of respondents agreed that local BC awareness campaigns are sufficient, whereas a large percentage of study participants evaluated awareness of BC in society as average (46%) or weak (42%). Moreover, only 19% of the participants were aware of BC screening programs in Saudi Arabia. On the other hand, a Saudi survey to assess knowledge about BC and mammography screening that included women in all regions of the kingdom, the majority aged between 21 and 40 years, showed that BC awareness activities and campaigns in the country are widespread, as approximately 80% of the respondents recognized them (Hagi et al., 2013). This noticeable difference in results between that study and ours was expected, indicating that BC awareness activities and campaigns in the kingdom are usually directed toward women with limited involvement of men. However, a clear description of a national BC screening program is lacking. In 2007, the first nationwide BC screening center was non-governmental and based in Riyadh city. Abulkhair et al., (2010) found that of 1215 women who were screened in this center, only 16 cases were detected. The first published report of a governmental BC screening program concerned a breast cancer screening program pilot study in the Al-Qassim region (Akhtar et al., 2010). In 2015, the Saudi MOH initiated a nationwide campaign to raise awareness about early screening for BC; however, the process for seeking BC screening is not clear (Saudi Ministry of Health). 

This knowledge assessment about mammography among men provides insights into the necessity of establishing more personalized education and awareness strategies about BC and mammography to all family members, males in particular. More active involvement of males in BC-related activities is recommended. In parallel with this effort, additional awareness campaigns that are designed to raise public awareness about BC and mammography screening in general, and in males in particular, and that highlight the importance of early detection of BC as essential for more effective outcomes.

Our study has some limitations. First, the results may not be generalizable to all men living in Saudi Arabia. However, different data collection methods were done to reach men and women from different geographical areas, educational levels, occupational and age groups. Second, these data may have been affected by reporting bias, as they were collected from self-reported questionnaires.

In conclusions, these results indicate a noticeable lack of knowledge about mammography among Saudi men. Nonetheless, the participants expressed positive behavior in recommending mammography screening to female relatives. This positive behavior was significantly influenced by their knowledge of mammography, age, educational level, and positive family history of BC. Additional awareness and educational programs designed for males to familiarize them with BC and its screening modalities are of great importance. Establishing national programs and educational campaigns for men to explain the benefits of screening and access to free mammography is essential for raising awareness about BC screening and increasing mammography rates among women in Saudi Arabia.

## Author Contribution Statement

LM and NF analyzed the data, interpreted the results, and critically reviewed the manuscript. RA designed the study, oversaw data collection, and critically revised the manuscript. RG wrote the manuscript and critically revised it.. All authors have critically reviewed and approved the final draft and are responsible for the content of the manuscript.
